# Screening of the shared pathogenic genes of ulcerative colitis and colorectal cancer by integrated bioinformatics analysis

**DOI:** 10.1111/jcmm.17878

**Published:** 2023-07-26

**Authors:** Xu Shi, Jun Yu, Chen Lu, Qian Luo, Caihong Xu, Jie Li, Wei Wang

**Affiliations:** ^1^ Department of Orthopaedics The Affiliated People's Hospital of Jiangsu University Zhenjiang China; ^2^ Department of Paediatrics Affiliated Hospital of Nanjing University of Chinese Medicine, Taicang Hospital of Traditional Chinese Medicine Taicang China; ^3^ Department of General Surgery Siyang Hospital Suqian China; ^4^ Department of Obstetrics and Gynaecology Nanjing Tongren Hospital, School of Medicine, Southeast University Nanjing China; ^5^ Department of Clinical Laboratory Lianshui County People's Hospital Huai'an China

**Keywords:** colorectal cancer, immune‐related genes, integrated bioinformatics analysis, machine learning, the shared pathogenic genes, ulcerative colitis

## Abstract

Ulcerative colitis (UC) is one of the high‐risk pathogenic factors for colorectal cancer (CRC). However, the shared gene and signalling mechanisms between UC and CRC remain unclear. The goal of this study was to delve more into the probable causal relationship between UC and CRC. CRC and UC datasets were downloaded from the Gene Expression Omnibus database. Using R software and Perl, differentially expressed genes (DEGs) in both UC and CRC tissues were re‐annotated and screened. The biological activities and signalling pathways involved in DEGs were investigated using Gene Ontology and Kyoto Encyclopedia of Genes and Genomes enrichment analyses. The STRING database and Cytoscape software were used to construct the gene interaction network. A total of 384 DEGs were selected for further investigation, and functional analysis revealed that inflammatory and immunological responses were crucial in the development of the two diseases. Moreover, the top 15 key genes involved in the UC and CRC were screened using cytoHubba, including IL1B, CXCL10, CCL20, MMP9, ICAM1, CCL4, CXCR1, MMP3, TLR2, PTGS2, IL1RN, IL6, COL1A2, TIMP1 and CXCL1. The identification of these genes in the present study may provide a novel perspective for the prediction, prevention and personalized medicine of UC and CRC patients.

## 1 INTRODUCTION

Colorectal cancer (CRC) is one of the most prevalent cancers globally, with approximately 1,148,000 new cases and more than 576,000 deaths each year.^1^ Numerous factors, including environmental and genetic factors, contribute to the aetiology and pathophysiology of CRC.^2^, ^3^, ^4^ Furthermore, several high‐risk factors, particularly ulcerative colitis (UC), are the leading causes of CRC.^5^, ^6^, ^7^, ^8^, ^9^ Thus, a thorough investigation of the mechanisms underlying the onset and progression of CRC is crucial for the detection and treatment of this disease.

UC is a chronic, non‐specific inflammatory disease of the gut. The lesions are common in the colon and rectum.^10^, ^11^, ^12^ The prevalence of UC has increased in recent years. Environmental causes, genetic factors and immune system abnormalities are often associated with UC.^13^, ^14^, ^15^, ^16^ A significant number of immune cells, such as dispersed neutrophils, lymphocytes, plasma cells and eosinophils, infiltrate the lesions, especially during an active illness.^17^, ^18^, ^19^, ^20^

Previous studies have demonstrated that patients with long‐standing UC (>20 years) have a higher risk for CRC.^21^, ^22^, ^23^ UC stimulates the growth of CRC via the inflammation‐cancer pathway, which is distinct from the typical adenoma‐adenocarcinoma pathway. However, the precise molecular mechanism through which UC promotes the development of CRC remains unknown. Thus, a comprehensive analysis of the molecular mechanism by which UC promotes the incidence of CRC is critical for the early detection and precise treatment of CRC.

The present study examined UC and CRC data from a high‐throughput sequencing (HTS) chip. A total of 384 genes (197 up‐regulated and 187 down‐regulated genes in both UC and CRC) involved in biological processes and signalling pathways were subjected to a gene ontology (GO) and Kyoto Encyclopedia of Genes and Genomes (KEGG) enrichment analyses. Various protein interaction networks were built to investigate the connections between these genes. Furthermore, key genes that may play crucial roles in UC and CRC were identified, and their functions and relationships were explored. This study provides new insights into the development of UC and CRC, as well as novel biomarkers for the early detection and treatment of CRC.

## 2 MATERIALS AND METHODS

### 2.1 Data acquisition

The RNA sequencing (RNA‐seq) data were retrieved from GSE87466 (GPL13158, 21 normal tissues and 87 UC tissues) and GSE87211 (GPL13497, 160 normal intestinal mucosa tissues and 203 CRC tissues) databases in the Gene Expression Omnibus (GEO) database (https://www.ncbi.nlm.nih.gov/geo/). GSE87466 includes normal intestinal mucosa tissues and UC tissues while GSE87211 contained normal and malignant CRC samples.

### 2.2 Identification of differentially expressed genes (DEGs)

Perl was used to re‐annotate data from GSE87211 and GSE87466 datasets. The reannotated data was subjected to differential expression analysis using the R packages limma, pheatmap, and ggplot2, and the results were presented as heat maps and volcano maps.

### 2.3 Identification of DEGs in UC and CRC

The R package VennDiagram was used to further investigate the DEGs in each GEO dataset. A total of 197 genes were up‐regulated and 187 genes were down‐regulated in both GSE87211 and GSE87466.

### 2.4 GO and KEGG enrichment analysis

R packages colorspace, stringi, ggplot2, circlize, RcolorBrewer and ggpubr were used for GO and KEGG enrichment analysis of the target genes to investigate the biological activities and signalling pathways associated with target genes.

### 2.5 Construction of protein–protein interaction (PPI) network

Using the STRING database (https://cn.string‐db.org/), a PPI network containing 384 genes was predicted and created. The PPI network was subsequently tested and the 3D bubble model was generated based on the screening criteria (minimum necessary interaction score = 0.700 and conceal unconnected nodes in the network).

### 2.6 Construction of co‐expression network

The co‐expression relationships of the 384 genes were analysed using Perl, and their co‐expression networks were visualized using Cytoscape software. Furthermore, the plugin MCODE in Cytoscape software was utilized to further examine the protein interaction sub‐network, and the screening settings were degree cut‐off = 2 and node score cutoff = 0.2.

### 2.7 Identification of key genes

The plugin cytoHubba in Cytoscape was used to score nodes of DEGs, and key genes with the highest node score were selected.

### 2.8 GeneMANIA analysis

The GeneMANIA database (http://genemania.org/) was utilized to further investigate the biological roles of each key gene and to forecast the genes that interact most closely with the key genes.

### 2.9 Tissue collection, extraction and quantitative real‐time fluorescence PCR

Cancer tissues and associated normal tissues were taken from colon cancer patients with a history of UC at The Second Affiliated Hospital of Nanjing Medical University, and each patient completed an informed permission form. The collection of human specimens was approved by the Ethics Committee of The Second Affiliated Hospital of Nanjing Medical University (approval no. [2019]‐KY‐121). The total RNA was isolated from tissues using Trizol (Life Technologies, Thermo Fisher, USA). HiScript (Vazyme, R323‐01, China) is used for reverse transcription. SYBR GREEN (Vazyme, Q321‐02, China) and LightCycler (Roche, USA) were used to perform real‐time fluorescence quantitative PCR. GAPDH was employed as a reference control. The supplement file contains primer sequences (Table S1).

### 2.10 Statistical analysis

All statistical analyses were conducted utilizing R software. P‐values ≤0.05 were considered statistically significant.

## 3 RESULTS

### 3.1 Identification of DEGs in UC and CRC

**FIGURE 1:**
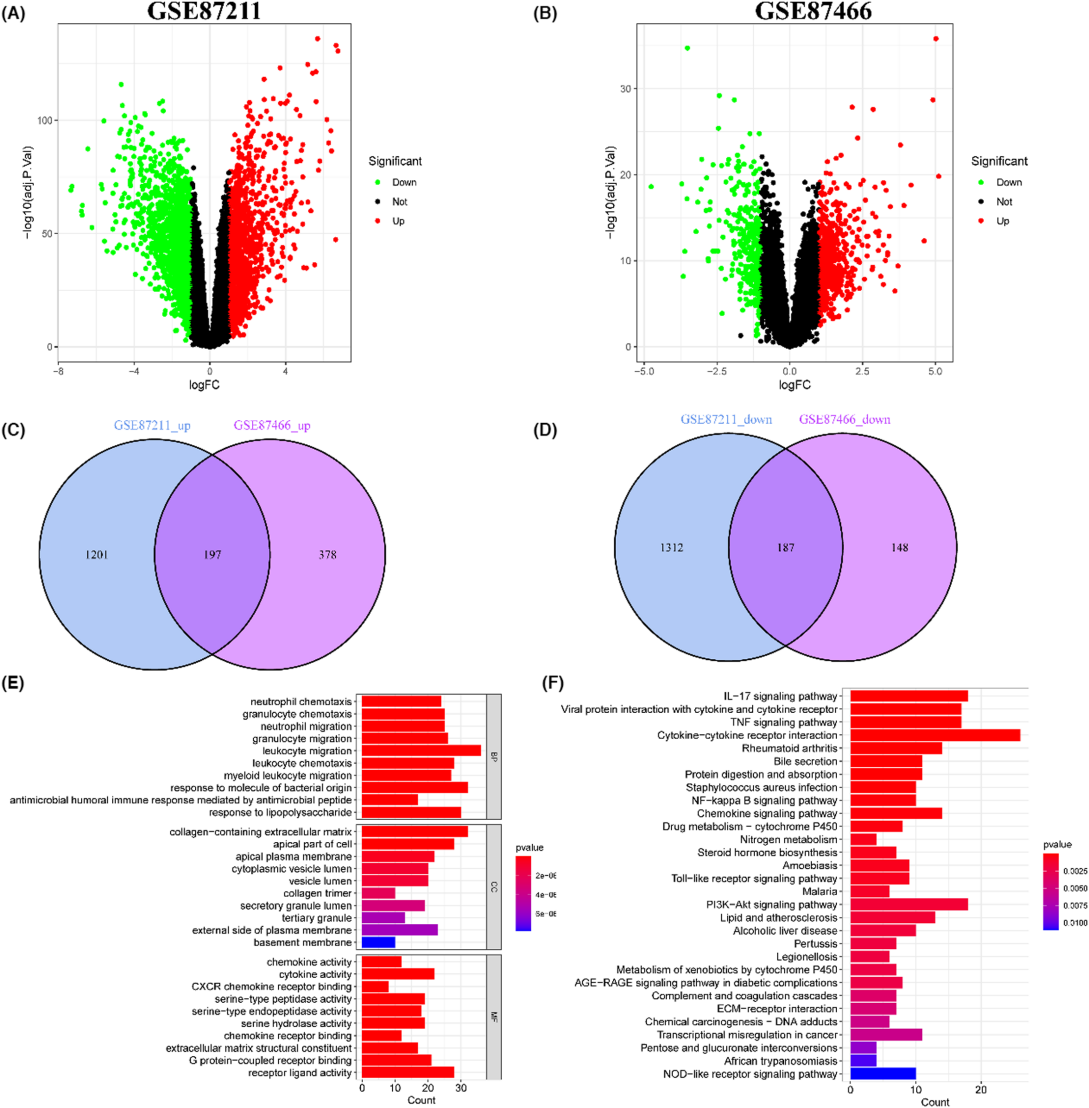
Identification of differentially expressed genes (DEGs) in GSE87211 and GSE87466 datasets. (A, B) DEGs in colorectal cancer (CRC) tissues (GSE87211) and ulcerative colitis (UC) tissues (GSE87466). (C, D) Significantly up‐regulated or down‐regulated DEGs in both GSE87211 and GSE87466 datasets. (E) GO functional analysis of DEGs. (F) KEGG pathway enrichment analysis of DEGs.

The RNA‐seq data were downloaded from GSE87211 for re‐annotation in the GEO database, including 160 normal intestinal mucosa tissues and 203 CRC tissues, and 21,713 genes were identified. Then, differential expression analysis performed based on the |log2 fold change (FC)| ≥ 1 and *p* ≤ 0.05 filter parameters, and 1398 up‐regulated and 1499 down‐regulated genes were identified in CRC tissues **(**Figure 1A **)**. In addition, the sequencing data were downloaded from GSE87466 for re‐annotation in the GEO database, including 21 normal tissues and 87 UC tissues, and 19,918 genes were identified. Then, differential expression analysis was performed based on |log2 FC| ≥ 1 and P ≤ 0.05 filter parameters, 575 up‐regulated and 335 down‐regulated genes were identified in UC tissues **(**Figure 1B **)**. The up‐regulated genes in GSE87211 and GSE87466 datasets were intersected, and 197 genes were obtained **(**Figure 1C **)**. The down‐regulated genes in GSE87211 and GSE87466 datasets were intersected, and 187 genes were obtained **(**Figure 1D **)**. Finally, 384 DEGs were identified in both UC and CRC tissues **(**Supplementary file 1**)**.

### 3.2 Go and KEGG enrichment analyses

GO and KEGG enrichment analyses were performed to identify the biological roles and signalling pathways associated with the 384 DEGs. GO analysis revealed that most DEGs were enriched in leukocyte migration, granulocyte migration, receptor‐ligand activity, myeloid leukocyte migration, etc. **(**Figure 1E **)**. KEGG enrichment analysis showed that most DEGs were primarily enriched in the interleukin 17 (IL‐17) signalling pathway, cytokine‐cytokine receptor interaction, phosphoinositide 3‐kinase (PI3K)‐serine/threonine kinase (Akt) signalling pathway, tumour necrosis factor (TNF) signalling pathway, nuclear factor‐kappa B (NF‐κB) signalling pathway, among others. **(**Figure 1F **)**.

### 3.3 Construction of the co‐expression network

**FIGURE 2:**
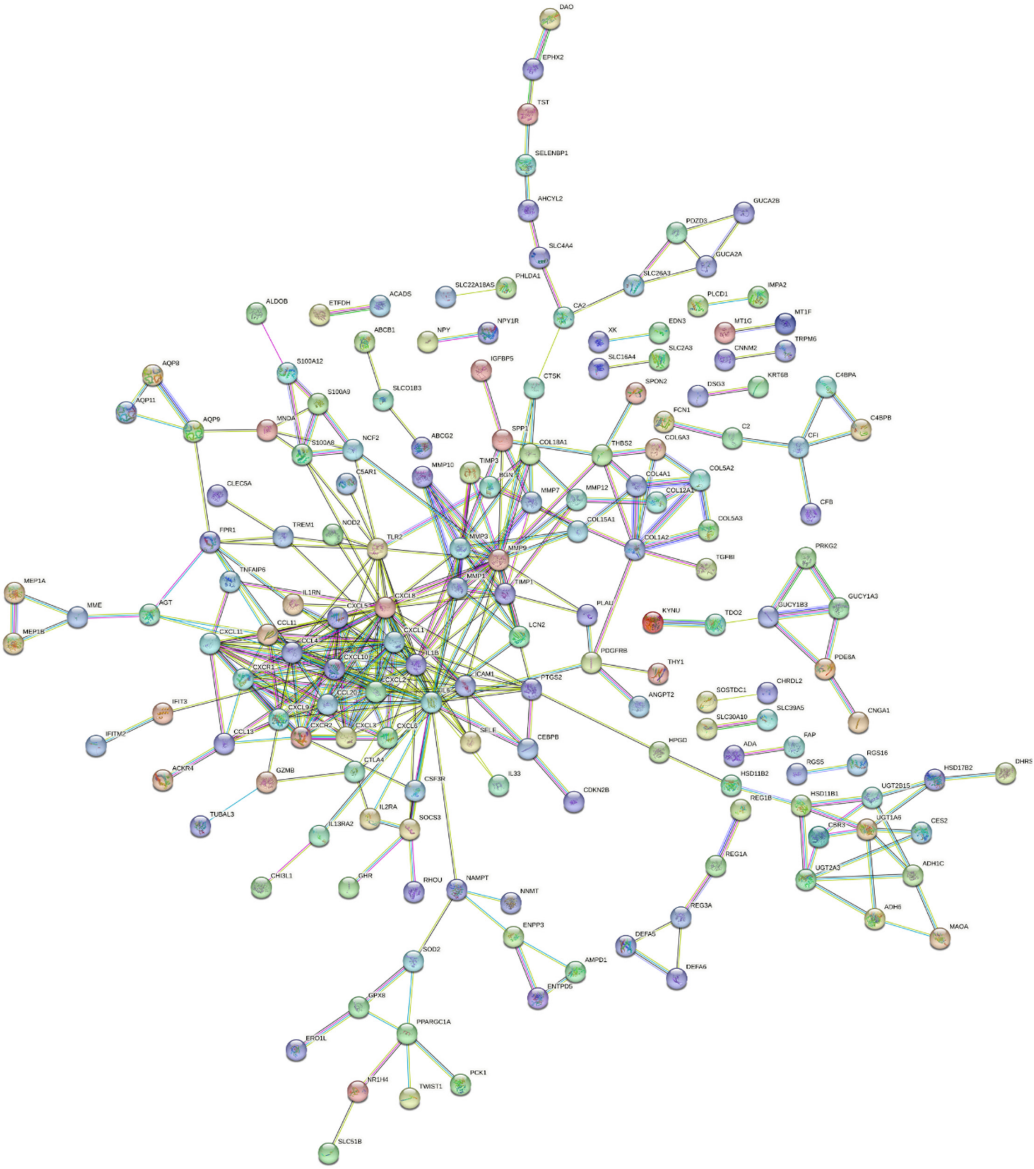
Protein–protein interaction network based on the STRING database.

**FIGURE 3:**
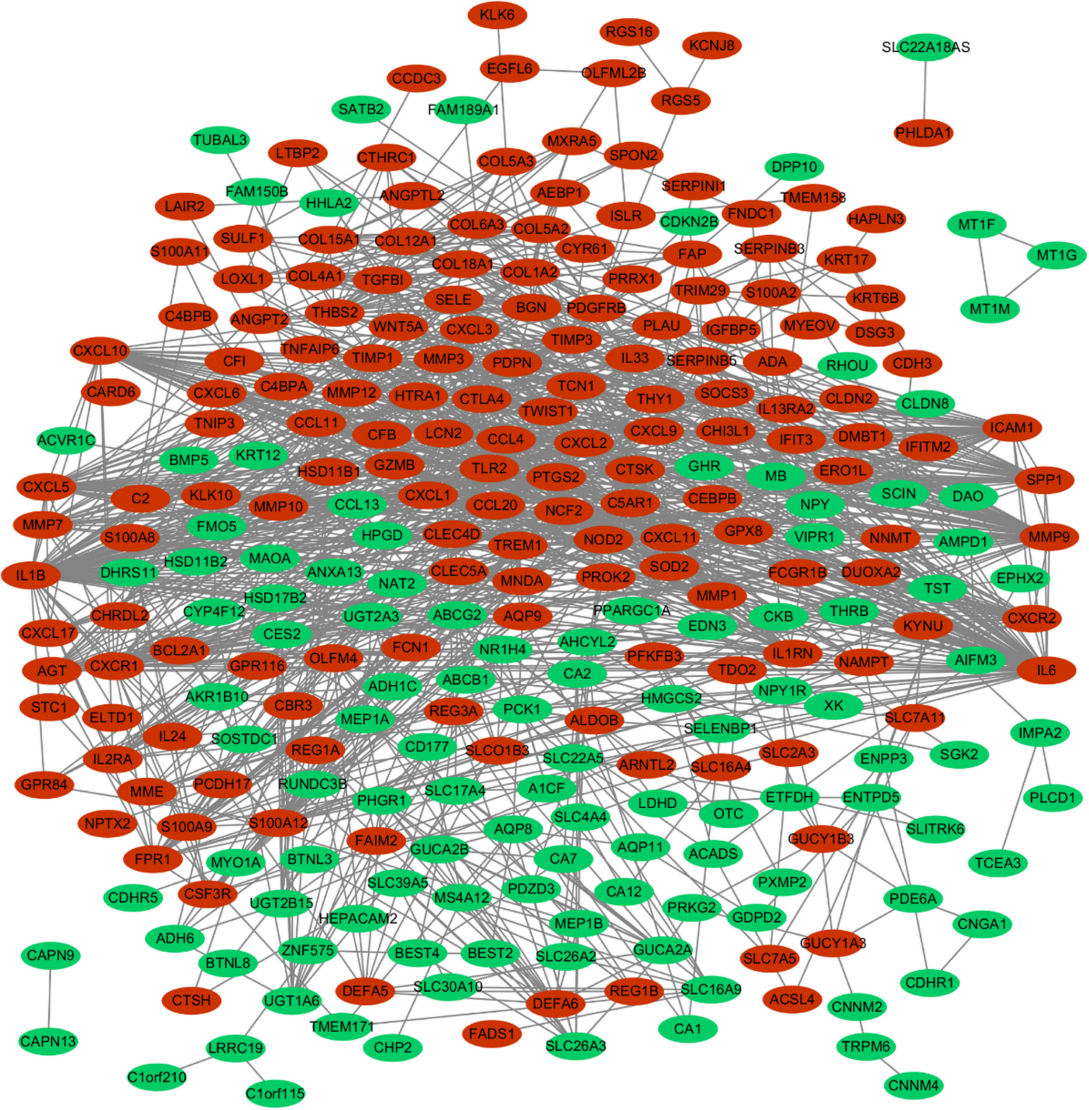
Gene co‐expression network analysis.

**FIGURE 4:**
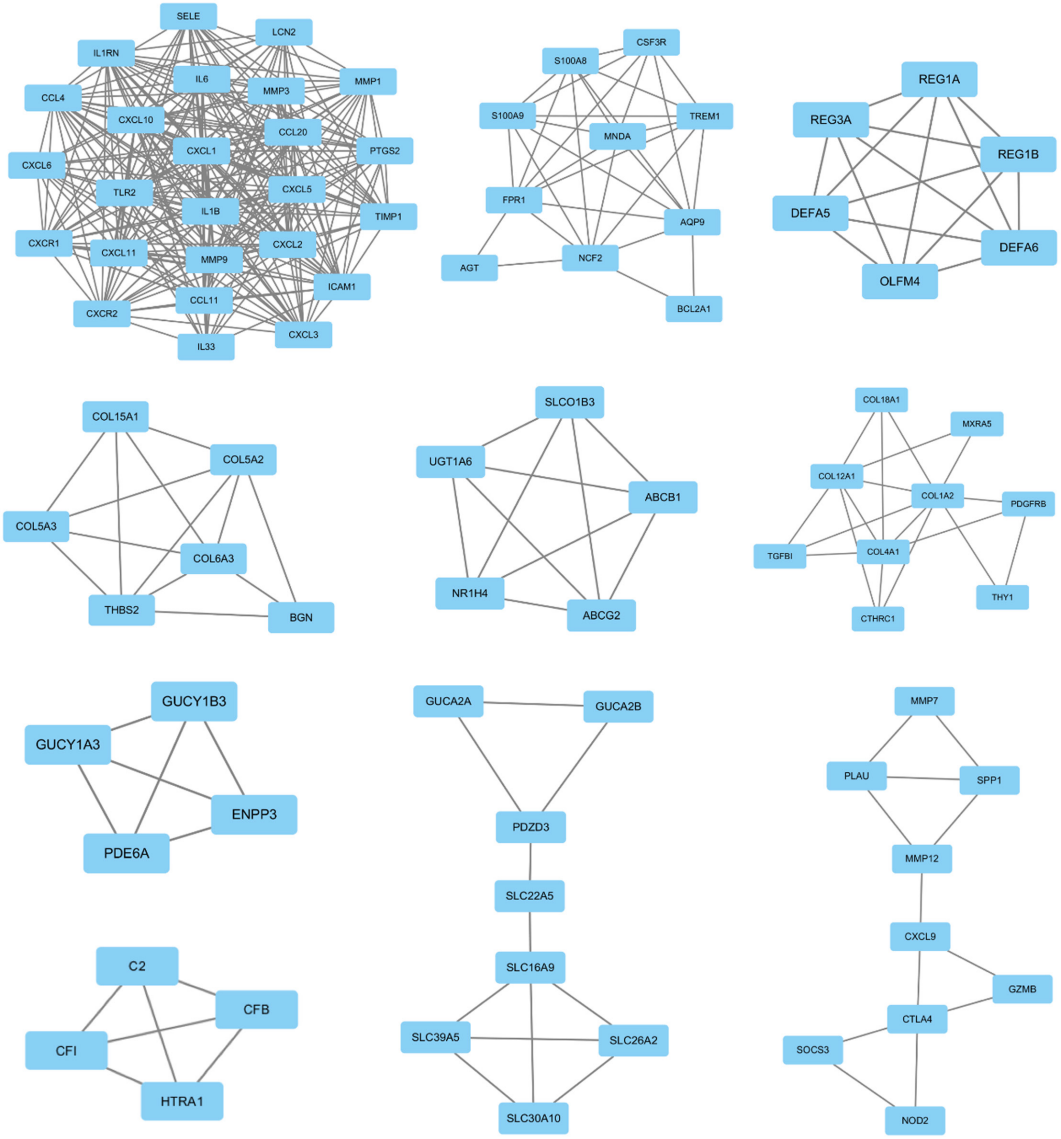
Protein–protein interaction sub‐network based on Cytoscape software.

The STRING database was utilized to build the PPI network between the 384 DEGs to further investigate their connection. The STRING database was used to calculate the interaction score between these proteins. The interacting proteins were screened and the PPI network was established with the minimum required interaction score ≥0.700 **(**Figure 2 **)**. Furthermore, the PPI network was visualized using Cytoscape software **(**Figure 3 **)**. The MCODE plugin in Cytoscape was used to identify the clusters in the PPI network based on the screening settings (degree cutoff = 2, node score cutoff = 0.2) **(**Figure 4 **)**.

### 3.4 Identification of key genes in UC and CRC

**FIGURE 5:**
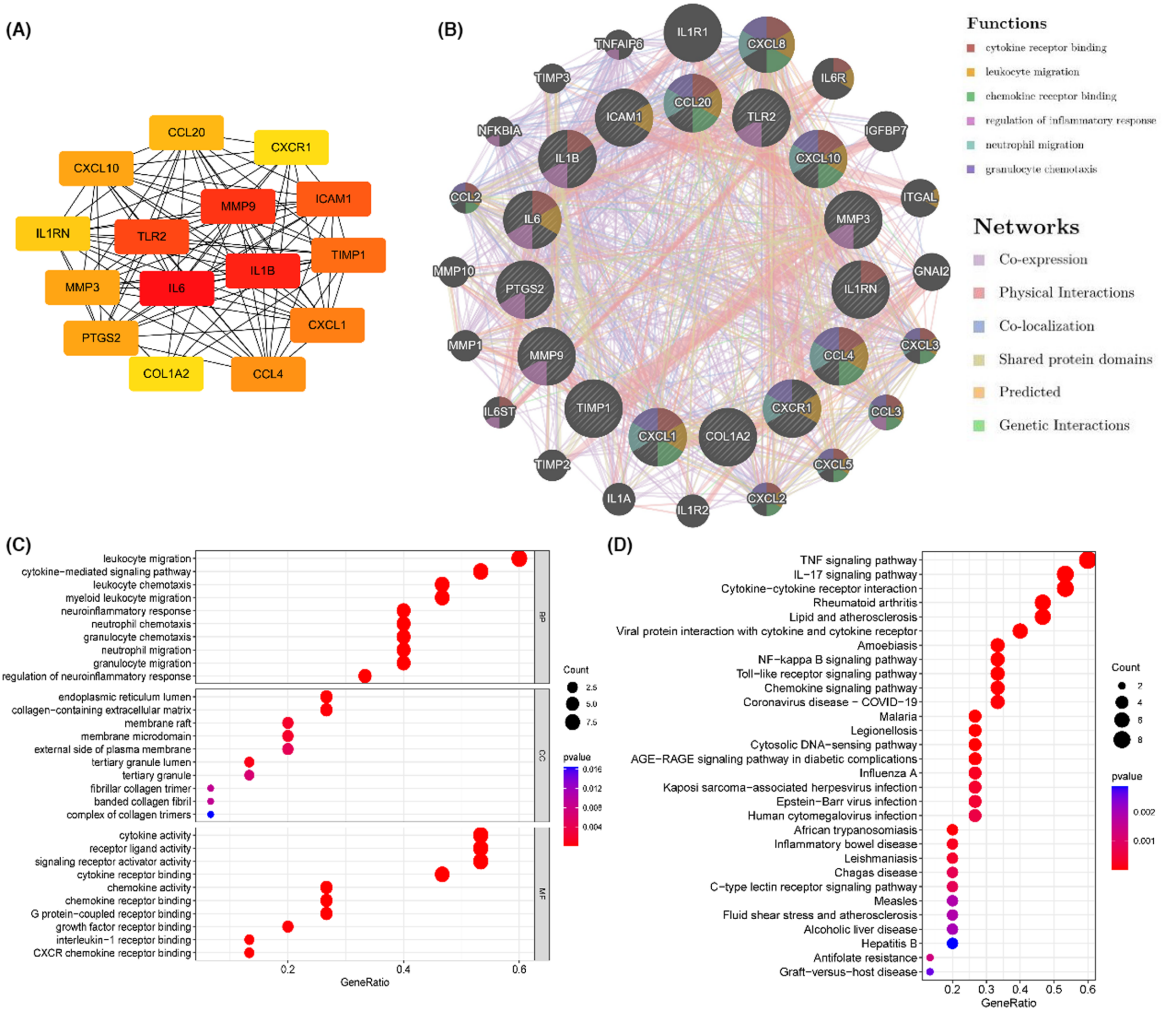
Identification of key genes in UC and CRC. (A) Co‐expression of key genes based on the Cytoscape plugin cytoHubba. (B) Co‐expression of key genes based on the GeneMANIA database. (C) GO functional analysis. (D) KEGG pathway enrichment analysis.

The Cytoscape plugin cytoHubba was applied to score the nodes of DEGs and the top 15 key genes with the highest node score were screened, including IL1B, CXCL10, CCL20, MMP9, ICAM1, CCL4, CXCR1, MMP3, TLR2, PTGS2, IL1RN, IL6, COL1A2, TIMP1 and CXCL1 **(**Figure 5A **)**. The GeneMANIA database was utilized to create an interaction network map of the key genes to further investigate their relationship with potential biological activities **(**Figure 5B **)**. The results revealed that the top 15 key genes were involved in multiple biological functions, such as cytokine receptor binding, leukocyte migration, chemokine receptor binding and regulation of inflammatory response.

### 3.5 GO and KEGG enrichment analysis of the top 15 key genes

GO and KEGG enrichment analyses were performed to identify the biological roles and signalling pathways related to the top 15 key genes. GO analysis showed that the top 15 key genes were enriched in leukocyte migration, cytokine‐mediated signalling pathway, cytokine receptor binding, signalling receptor activator activity, etc. **(**Figure 5C **)**. KEGG analysis revealed that the top 15 key genes were primarily enriched in TNF signalling pathway, IL‐17 signalling pathway, Toll‐like receptor (TLR) signalling pathway, NF‐κB signalling pathway, etc. **(**Figure 5D **)**.

### 3.6 Validation of key genes

**FIGURE 6:**
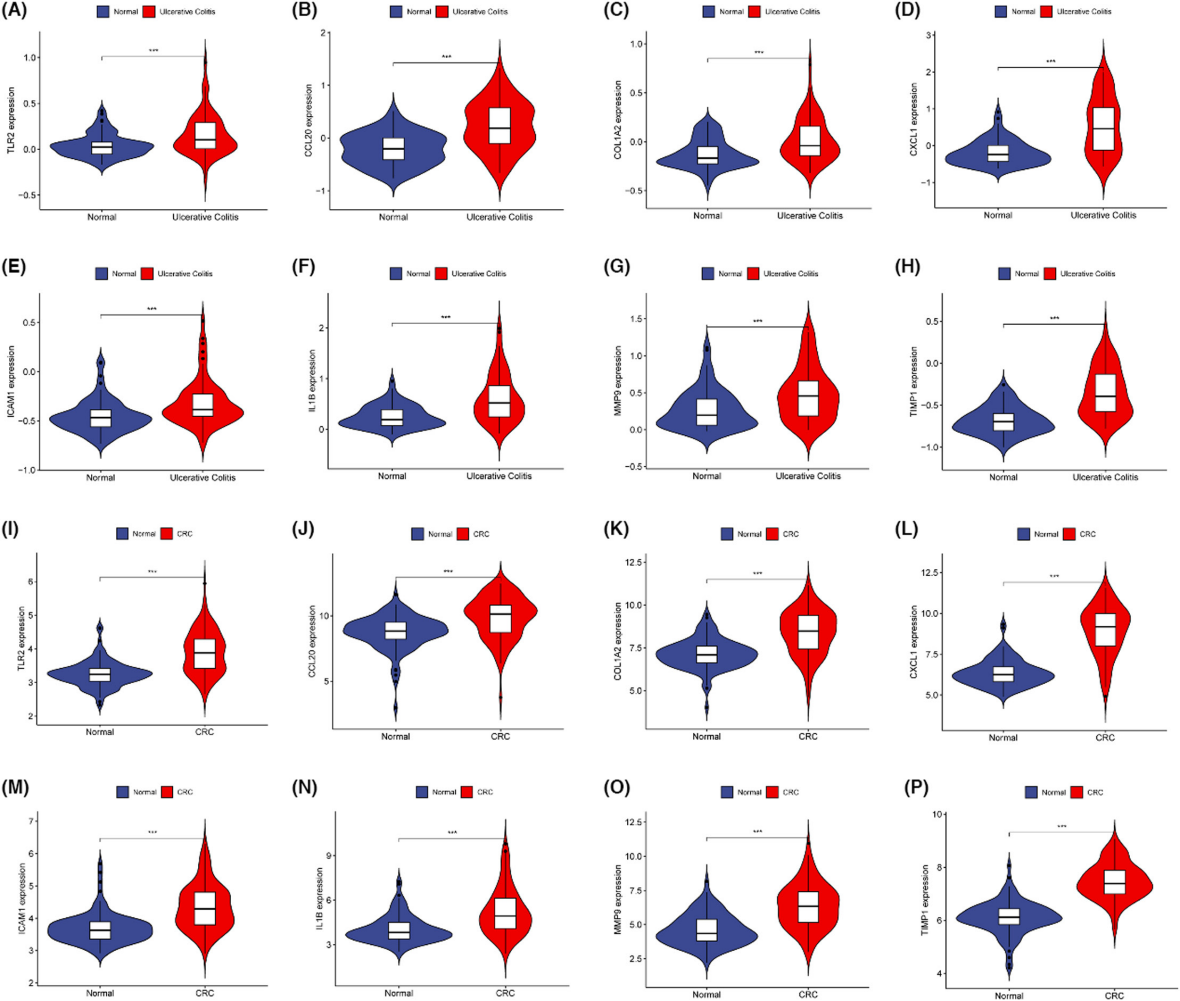
Validation of key genes. (A–H) Key genes were significantly up‐regulated in UC tissues (GSE11223). (I–P) Key genes were significantly up‐regulated in CRC tissues (GSE44076).

**FIGURE 7:**
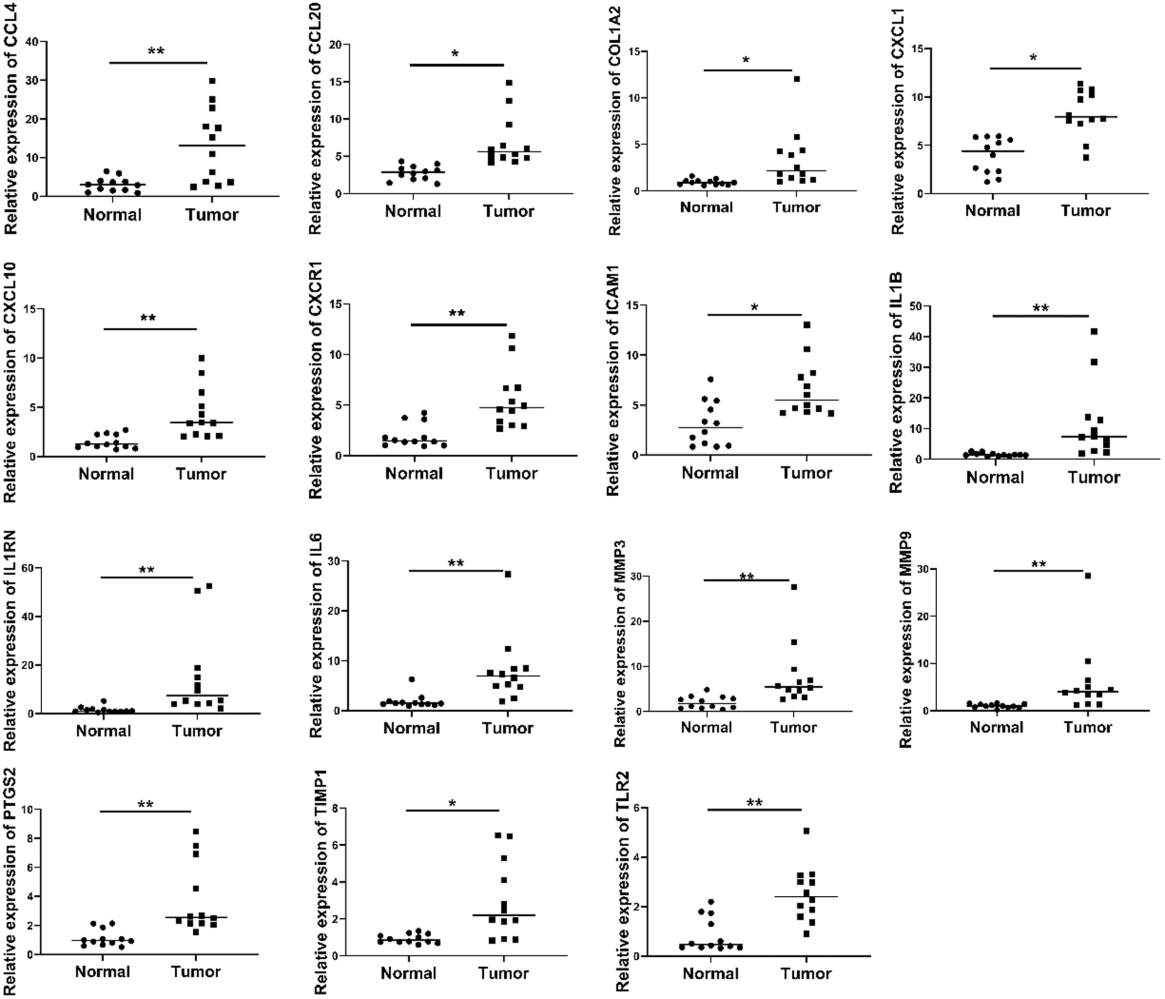
qRT‐PCR was performed to evaluate the expression of 15 key genes in the tissues of colorectal patients with ulcerative colitis.

To verify whether the key genes screened in the experimental sets (GSE87211 and GSE87466) were generalized, RNA‐seq data for CRC (GSE44076) and UC (GSE11223) were retrieved from the GEO database as validation sets. Differential expression analysis of the validation sets revealed that the top 15 key genes were significantly expressed in both UC and CRC tissues compared with normal intestinal mucosa **(**Figure 6 and Figure S1 **)**. Additionally, using qRT‐PCR, we examined the expression levels of 15 core genes in the colorectal tissues of individuals with a history of UC. We found that, as compared to normal tissues, all 15 core genes were substantially strongly expressed in cancer tissues (Figure 7).

## 4 DISCUSSION

CRC is the third most common cancer type worldwide. The development of molecular targeted treatment and immunotherapy has improved the overall survival of patients with CRC.^24^, ^25^, ^26^ However, the overall survival rate of individuals with recurrent, metastatic, or advanced CRC remains poor.^27^, ^28^, ^29^ Therefore, early diagnosis and treatment of CRC are essential to improve the overall survival rate for CRC patients. Several factors are associated with CRC pathogenesis. For instance, UC is a well‐known high‐risk factor for CRC. It is a chronic non‐specific intestinal inflammatory illness characterized by the infiltration of a significant number of immune cells at the lesion site, which results in changes in the immunological microenvironment.^19^, ^30^, ^31^ It is well established that people with long‐standing UC have a higher risk for CRC. Therefore, a better understanding of the transition from UC and CRC may provide insights into the shared molecular mechanisms to identify novel biomarkers for UC and CRC.

The combined analysis of multiple microarrays in the GEO database identified 197 up‐regulated and 187 down‐regulated genes in both UC and CRC tissues. Furthermore, using the Cytoscape plugin cytoHubba, we scored and screened 384 genes and retrieved the top 15 key genes, including IL1B, CXCL10, CCL20, MMP9, ICAM1, CCL4, CXCR1, MMP3, TLR2, PTGS2, IL1RN, IL6, COL1A2, TIMP1 and CXCL1.

These top 15 key genes may play essential roles in UC and CRC. C‐X‐C motif chemokine ligand 1 (CXCL1) and CXCL10 are members of the CXC chemokine subfamily,^32^, ^33^, ^34^ C‐C motif chemokine ligand 4 (CCL4) and CCL20 are members of the CC chemokine subfamily,^35^, ^36^, ^37^, ^38^ and C‐X‐C motif chemokine receptor 1 (CXCR1) is a receptor for the CXC subfamily.^39^, ^40^, ^41^ It has been shown that members of CXC chemokines are chemotactic to neutrophils, and CC chemokines are chemotactic to monocyte and lymphocyte subsets. The aberrant expression of chemokines and their receptors in UC and CRC tissues suggests that immune cells play a key role in the transformation of both diseases. Members of the IL‐1 cytokine family, IL‐1B and IL‐1RN, as well as IL‐6, play significant roles in the cellular inflammatory response.^42^, ^43^, ^44^, ^45^, ^46^, ^47^, ^48^ The aberrant expression of IL‐1B, IL‐1RN, and IL‐6 indicates that cellular inflammation is related to UC and CRC. The matrix metalloproteinases (MMP) family includes MMP3 and MMP9,^49^ and tissue inhibitor matrix metalloproteinase 1 (TIMP1) is an MMP family natural inhibitor.^50^, ^51^ MMP family members play crucial roles in UC and CRC.^52^, ^53^, ^54^, ^55^ The present study discovered the aberrant expression of MMP family members in the UC and CRC transition, indicating that MMP family members may be crucial molecules in the inflammation‐cancer transformation process. Intercellular adhesion molecule 1 (ICAM1) is a glycoprotein that is usually expressed on endothelial cells and immune system cells.^56^, ^57^ TLR2 belongs to the TLR family, which is involved in pathogen identification and innate immune activation.^58^ Prostaglandin endoperoxide synthase isozyme 2 (PTGS2) has been found to induce inflammation.^59^ The collagen type I alpha 2 chain (COL1A2) gene encodes the pro‐alpha2 chain of type I collagen and has been linked to the onset and progression of UC and CRC.^60^ These essential genes are strongly associated with inflammatory and immunological responses, implying that these responses may play a crucial role in the process of UC and CRC transition. Although some studies have shown that these key genes contribute to the occurrence and progression of UC or CRC, their exact function and underlying molecular mechanism in the UC and CRC transition remain elusive and are worthy of investigation.

However, this study has several limitations. First, our findings are based only on bioinformatics analysis and lack experimental validation. Second, we merely hypothesized the biological roles and signalling pathways that could be implicated in abnormally expressed genes in UC and CRC, but the underlying mechanisms were not elucidated. Finally, the differential expression of key genes was only verified in several HTS chips from the GEO database, and therefore more clinical data are needed for further validation.

In summary, the current study investigated key molecules and examined biological activities and signalling pathways associated with the UC and CRC. This study did not focus on the single‐disease analysis of CRC but explored essential genes involved in the double‐disease transformation process. Firstly, bioinformatics analysis was employed to screen the pathogenic genes involved in UC and CRC. The findings of this study provide new scientific insights into the investigation of potential molecular pathways between UC and CRC, which is useful for the practice of PPPM in UC and CRC. Second, identifying key genes in the course of UC and CRC is useful for early prognosis and prevention of CRC. Our findings may help determine normal reference values and diagnostic thresholds of key genes in CRC patients in clinical practice to aid in the prevention of UC and CRC. Dynamic monitoring of core gene expression levels in UC patients may aid in the detection of CRC at an early stage for timely and accurate treatment. Furthermore, DEGs were strongly associated with inflammatory and immunological responses, which might aid in the prevention and treatment of UC and CRC. Finally, targeted drug therapy based on core genes is a highly promising approach. Our findings revealed novel biomarkers for the early prevention and treatment of UC and CRC.

## AUTHOR CONTRIBUTIONS

**Xu Shi:** Conceptualization (equal); data curation (equal). **Jun Yu:** Investigation (equal); methodology (equal); validation (equal). **Chen Lu:** Resources (equal); software (equal); supervision (equal). **Qian Luo:** Formal analysis (equal); investigation (equal); methodology (equal); project administration (equal). **Caihong Xu:** Formal analysis (equal); validation (equal); visualization (equal); writing – review and editing (equal). **Jie Li:** Conceptualization (equal); data curation (equal); writing – original draft (equal). **Wei Wang:** Data curation (equal); formal analysis (equal); methodology (equal); resources (equal).

## FUNDING INFORMATION

The authors declare that no funds, grants, or other support were received during the preparation of this manuscript.

## CONFLICT OF INTEREST STATEMENT

The authors declare that they have no competing interests.

## ETHICS STATEMENT

The declaration of Helsinki's guiding principles were followed in conducting this study. Informed consent was obtained from all the participants. The Medical Ethics Committee of the Second Affiliated Hospital of Nanjing Medical University gave its approval to each experimental procedure.

## CONSENT

Not applicable.

## Supporting information


Figure S1.

Table S1.

## Data Availability

The datasets analysed during the current study are available in the GEO databases (https://www.ncbi.nlm.nih.gov/geo/). The analyses methods and used packages are illustrated in the “Materials and methods” section. All other R code and analyses are available from the corresponding author upon request.
