# Supplementary figures and images for: Screening of the shared pathogenic genes of ulcerative colitis and colorectal cancer by integrated bioinformatics analysis
Source: J Cell Mol Med. 2023 Jul 26;28(5):e17878. doi: 10.1111/jcmm.17878 (PMC10902564; doi:10.1111/jcmm.17878)

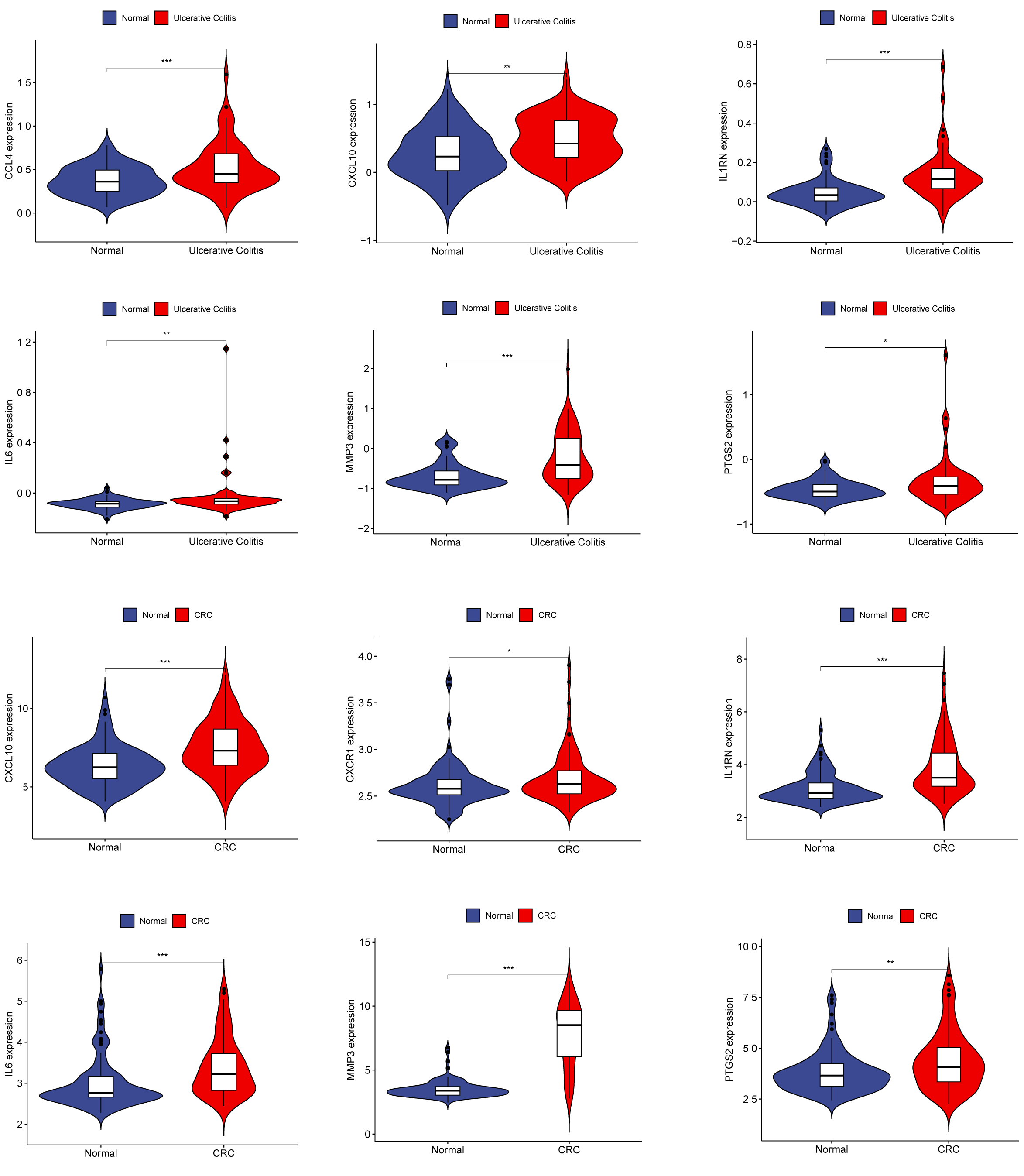

Supplement: Supplementary file 1 — Figure S1. [file JCMM-28-e17878-s002.tif]
